# Obstacles to using the safe surgery checklist: Perspectives of first-line personnel

**DOI:** 10.1177/20503121241278229

**Published:** 2024-09-18

**Authors:** Marianne Palm, Geir Sverre Braut

**Affiliations:** 1 Oslo University Hospital, Oslo, Norway; 2 Stavanger University Hospital, Stavanger, Norway; 3 HVL Business School, Western Norway University of Applied Sciences, Bergen, Norway

**Keywords:** Patient safety, safe surgery checklist, HazID method, system’s perspective, clinical safety management

## Abstract

**Objectives:**

The safe surgery checklist, presented by the World Health Organization in 2008, is an aid to performing surgical interventions safely. Research indicates that the use of checklists in clinical activities leads to a reduced number of adverse events. However, research suggests that the use of checklists differs between different institutions and even between units in the same organisation. The intention of this study is to identify factors regarded by the health personnel in ‘the sharp end’ as obstacles to using the checklist.

**Methods:**

The study has a qualitative, case-based design. It is performed by the Hazard Identification method, which is a method for revealing safety hazards based on workers’ experiences.

**Results:**

Obstacles were identified related to the content of the list, areas of use, distribution of responsibilities connected with the use of the list, and finally the organisation and management of safety efforts related to clinical activities. The use of checklists must be part of a system’s perspective, and deviations from checklists must be discussed in the organisation. The informants also claimed that checklists should be implemented for interventions located outside the operating theatres and for emergency treatments.

**Conclusions:**

Even though the majority of employees believe that checklists are necessary, many surrounding factors are perceived as obstacles to their use. Not least, site-specific factors may be revealed by use of the Hazard Identification method.

## Introduction

In 2008, The World Health Organization presented a checklist for use regarding surgical interventions. The checklist is an aid to performing surgical interventions safely according to sound professional practice. The checklist typically is divided into three parts: (I) Before induction of anaesthesia, (II) Before skin incision and (III) Before patient leaves operating room.^1^ The checklist’s points cover activities to ensure correct patient data, reviewing preparations, including positioning the patient on the table, presenting the team involved in the treatment, necessary postoperative reporting and follow-up, etc.

Data from several studies indicate that the use of checklists in clinical activities leads to a reduced number of adverse events with surgical interventions in hospitals.^2–10^

There is also data suggesting that the use of checklists differs between different institutions and even between units in the same organisation.^11^ This also applies to the hospital from which this study comes, despite previous studies performed locally that have demonstrated the importance of using checklists.^12,13^ It is also shown that when the checklist is not integrated into the risk management strategy of the hospital but is perceived as an ‘add on’, the fidelity in it is low.^14^ Recently, possible adjustments to the checklist have been suggested based on empirical findings.^15,16^

This study aims to identify obstacles perceived by the health personnel at the ‘sharp end’ of service delivery for using checklists as required by the hospital’s safety and quality system. It is limited to the use of checklists related to surgical procedures.

The study was performed at Oslo University Hospital and Stavanger University Hospital, which both have a diversity of surgical subspeciality units, offering programmes for specialisation of physicians in different branches of surgery, as well as education of specialised nurses in surgery, anaesthesiology, intensive care, and midwifery. Both hospitals have a broad portfolio of research in surgery and are teaching hospitals for nursing and medical students.

## Theory

A core principle in contemporary safety science is to identify causal chains showing how different factors influence the outcome of the working processes.^17^ In the 1990s, James Reason established a model showing how different types of defences, sometimes also called barriers, can stop possible hazards or dangers from developing into losses.^18^ A checklist for safe surgery is a tool to ensure that such defences are established and withheld in the operation theatre.

It is worth noting that the checklist should not be regarded as a defence by itself. The actions described in the checklist are the actual defences.^19^ Thus, lacking a checklist may not be isolated as a factor leading to incidents. However, previous research as cited above, has pointed out that using a checklist ensures that actions relevant to patient safety are more likely to be implemented. In addition, the active and predictable use of a checklist may also serve as a symbol, showing the institution’s total set of efforts to reduce threats to patient safety. However, caution should be taken to use checklists on simple and linear processes with high impact on safety, rather than extending the use of them to more complex processes where they may confuse and increase complexity more than enhance safety.^20,21^ Therefore, the use of a checklist should be perceived as a part of an active system for safety management.^22^

## Method

We chose a qualitative design based on case studies to identify factors regarded as obstacles to using a checklist in a clinical setting.^23^ As we were going to identify factors that could threaten patient safety, we decided to use the HazID method (an acronym for Hazard Identification). The core content of the method is focus group-like interviews. The approach used here was as described by Siddiqui et al.^24^ It is a generic method that can be adapted to the nature and particular challenges of the activities involved.^25,26^

The discussions in the focus groups were triggered by a single, open question, which is a common, initial step in HazIDs.^27^ The interview guide used in this specific study was not separately validated. This was regarded as not relevant because it could not give any sensible information. The participants were initially directly invited to describe and discuss their experiences using the checklist in their ordinary work. Based on how the discussions developed, the group leaders supplemented with questions on what was perceived as obstacles and facilitating factors for using the checklist. The group interviews lasted between 30 and 60 min, mostly depending on the number of participants in each group.

Many methods are available for identifying risk areas.^26^ Using one method does not exclude others. However, the HazID method appears well suited for use when it is important to involve groups of employees who work directly in production.^27^ The HazID method is a systematic method for assessing and identifying risk factors in a system or activity. Structured group interviews are performed with three employees directly connected with the tasks and procedures being considered. A certain safety-related topic is chosen, and the participants describe the ‘operation/task’. Then, keywords are presented, either by the participants themselves or by the group leaders, that indicate possible deviations from the normal practices or the expected standard, and risk factors are identified.^27^ When using the HazID method, risk factors are described in detail from a ‘present-day perspective’ by those who perform the services daily. In an ideal HazID process, the keywords are presented by the participants themselves. No specific validation for use in this project was performed due to the generic approach applied.^24–27^

The participants were all clinically working employees in different departments and units participating in surgical procedures in the two university hospitals. They had different professional affiliations. All were either doctors or nurses actively working in the operational theatre. Six interviews were conducted in the period from November 2021 to June 2023 with a total of 14 participants, of which 9 physicians (2 women, 7 men) and 5 nurses (3 women, 2 men), cf. Table 1.

**Table 1.** Demographics of the participants.

Age group	Female	Male
30–39	0	2
40–49	4	3
50–59	2	3

Only the group leaders (authors) and participants were present at the meeting. When finishing the fifth and sixth group there was scarcely any new information gained. It appeared that saturation had been achieved.

In preparation for the project, all heads of clinics where the checklist is in use were informed and invited to give feedback on the project plan. Thereafter, persons *not* engaged in the research project were responsible for inviting doctors and nurses to participate in the HazID groups. One of the invited dropped out before entering the HazID group due to upcoming working obligations.

Written information on the project was provided in the invitation and repeated at the beginning of the group discussions, when also oral consent was given in addition to the written consent given by initial e-mail. The documents containing names were deleted when the written reports from each focus group were accepted by the participants. No follow-up meetings were performed. All information was analysed in anonymised format as to names, sex, profession, and organisational affiliation.

The project was conducted according to requirements in Guidelines for Research Ethics in the Social Sciences and the Humanities.^28^ Participation was voluntary and based on consent from every participant. No personal data of special categories were collected, cf. General Data Protection Regulation (GDPR) article 9, no. 1.^29^ According to current national and European legislation related to data protection, the project was conferred with the data protection officer at both hospitals, who had no objections. Because patient data were not used, there was no need for further ethical approvals.

Both authors (respectively registered nurse and physician) were present at the interviews. Statements from the participants were recorded on paper. No electronic recording equipment was used during the interviews. After transcription, the interview records were presented to participants, who were invited to make corrections. Only suggestions for minor textual adjustments were received.

Thereafter, the written documentation was analysed by content analysis with open coding by both authors.^30^ As performed in this project, content analysis is an inductive approach where we search for patterns, enabling us to group the findings into particular themes or categories. The results or theories that emerge through this form of coding can thus generate new theories and/or hypotheses. If desired, these can later be used as deductive analysis by comparing new data to these.

## Results

Our approach, as described briefly above, leads us to the description of five themes and one set of suggestions presented by the interviewees, which shall be further considered here.

### The contents of the checklist

The content is perceived as relevant and important, but with the focus that the list must not be too long. One way of putting this in the group discussions was: ‘Everyone knows the questions, and if the list is too long, it may just become tiring’. However, another typical statement was. ‘If the list was not to be used, we would have overseen lots of important factors’. It thus appears that it is important to use checklists also for interventions/procedures, not merely surgical operations. However, the content must be adapted to the activity which also was commented by all groups. Especially related to acute interventions, checklists were regarded as important. Still, the interviewees commonly stated that the list should cover only a few important topics to avoid being too time-consuming.

About the item that deals with the presentation of the team working together, there was a very wide gap as to whether this was included in practice and whether the participants in the team saw this as an important point. In some cases, yes/no answers to questions were not considered comprehensive, and there was, therefore, a desire that the answers here should be better specified.

One of the interviewees mentioned that at one specific department, they had positive experiences performing slight adjustments to the checklist to fit their own purposes.

### Areas of use

In operating theatres, checklists for safe surgery are generally well incorporated. Because there is constant great development in medical treatment methods, this has led to much of the patient treatment being moved out of the operating theatres, for example, to special laboratories and other types of examination/treatment arenas.

In places outside the operating theatres, checklists are not used as much as in ordinary operating theatres. Thus, there is a greater possibility of unwanted incidents occurring. Activity at so-called ‘outstations’ also involves personnel without work connected to operating theatres and are therefore not as used to using checklists. Regarding interventions/procedures, it became clear that there was a need to use checklists within this field, but today, this use is not a fixed routine.

A special concern, presented by most of the groups, was related to busy emergency situations when, as stated in one of the groups: ‘The responsible surgeon often forgets to use it’.

### Distribution of responsibilities

The responsibility for completing and using checklists is placed on different persons/roles varying between the places that use them, even if they are used in acute situations. There is no uniform functioning system across different units. One possible point of failure presented by most groups was that: ‘Some persons in the team continue with their own thoughts and activities instead of focusing the reading of the list’.

At one of the hospitals, most wanted the operating room nurses to be the ones to be responsible for guiding through the checklist, but there are various reasons for this. Among the claims presented by the interviewees, we found expressions about nurses ‘having a marker handy’. They are the driving force. It is felt that they are the ones taking responsibility, etc. Some participants also believed that the operator should be responsible for reading the checklist. At the other hospital, the role of guiding through Phases 2 and 3 was unanimously placed on the surgeon.

Another point raised was that all important actors were not responsible for being present when checklists were to be reviewed. This was perceived as checklists not being a joint task with joint responsibility. Absence and little participation when the checklists were to be reviewed emerged as a challenge, and there was uncertainty about the completion quality when there was a change of guard during an intervention. When it came to using checklists at ‘outstations’, it emerged that staff felt that ‘nobody’ owned the patient, and therefore, this could be an explanation for checklists not being used.

### Organisation

The presentation round was a point that appeared to be highly debated. One particular concern presented several times was that: ‘The presentation round may disturb the rhythm in the work, especially in situations when we all already know each other well’. If the presentation round was carried out, this could lead to experiences of some being insulted for not being known by everyone, and it also emerged that the hierarchy was important in the form of younger nurses finding it difficult to ask to take the presentation round if there were older experienced doctors in the team.

If the presentation round was not taken, this led to frustration and uncertainty in some situations. The experience of working in a team differed from unit to unit. Still, it emerged that where the employees experienced working in a team, it was easier to agree on the use and importance of checklists. Typical expressions were that: ‘The check list makes a common agenda’ and ‘The reading of the list sets focus’.

The teams in the operating theatres are often made up of employees who belong to different departments/clinics. This emerged as a disadvantage in terms of creating good and secure relationships in the teams, in addition to the fact that this could also be a challenge for the distribution of responsibilities when it came to reviewing and completing checklists. According to the plan, the checklist must be registered in electronic patient record systems. This could not always be done, partly due to uncertainty about where it should be registered. In many cases, it was noted that checklists were not fully completed. The purpose of using checklists sometimes appeared unclear and varied among different units.

### Management

There is no system for monitoring when, where and how checklists are used, nor has it been determined who is responsible for them being used. The use of the checklist is documented in the case files connected with the operation. Still, data is not being further used for quality improvement or follow-up of the checklist. The employees claimed that training and registration of the checklist and interaction between leaders and employees were important points that managers had to address. In the group discussions, the employees called for increased responsibility and involvement from managers. Crucial in all groups was that: ‘Safety culture is a managerial responsibility’.

### Suggestions for improvement given by the interviewees

To ensure that checklists are used, they must be adapted to the type of intervention they are used against. This means that practice is also established for checklists for acute interventions. Furthermore, the checklists must not be too long. It must not take too long to check out the points, as this can determine whether checklists are prioritised when time is short.

Some of the points in the checklists can be answered with yes-no, but employees believe this is not good enough. Answers must be able to be specified, and in addition, it must be possible to write, for example, the time and dose for given drugs (in particular antibiotics) and specifications of any important messages for the postoperative course. A typical comment was: ‘It is not enough to answer yes or no. You have to specify a bit’.

Much patient treatment has now been moved out of the traditional operating rooms and into other arenas such as outpatient clinics, special laboratories and other treatment and examination rooms. Thus, the employees see that there may be an increased risk of unfortunate incidents and patient injuries if regular use of checklists is not applied also in these places.

When the checklists have been completed, and the treatment has ended, an improvement must be made to ensure that the data is recorded in the patient’s electronic medical record. It must also be decided who will take responsibility for confiscating this registration. The teams in the operating theatres often consist of personnel from different clinics and departments, which several believe is an obstacle to optimal collaboration. A shared sense of responsibility and a good team experience is needed. Therefore, they wish that the organisation of personnel could be rearranged.

The themes generated from the analysis are presented in Table 2.

**Table 2.** Themes generated in the study.

Themes	Subthemes
The contents of the checklist	• Covers relevant and important topics • Should not become too long • Should only cover important topics • Content adapted to the specific activity • Presentation of the team often not done
Areas of use	• Always used in the operating theatre • Often not used for procedures outside the operating theatre, which should be done
Distribution of responsibilities	• Responsibility for completing the checklist varies between the units/departments, especially at sites outside the operating theatre • Important actors may not be present when checklist is read • Especially for phase 3, there seemed to be unclear responsibility at some units at one of the hospitals
Organisation	• The need for the presentation round was debated, especially among experienced doctors • Commonly accepted that the presentation was important to reduce uncertainty and minimise frustration in the team • Uncertainty on how to document the use of the checklist
Management	• No procedure exists for a systematic monitoring of the use of the checklist • Need for an increased engagement from the managerial level
Suggestions for improvement	• The checklist must be adapted to the specific needs of every unit • The checklist must be kept short • The checklist must also be used outside the operating theatre • The use of the checklist must be better documented • The responsibility for reading the checklist must be unanimously placed, especially in phase 3

## Discussion

The experience from this study is that the HazID method, performed according to common practice, may be useful when aiming to reveal obstacles to safe working practices, not least such factors that are specific to any particular work site. The discussions in the focus groups in general went smooth after presenting the initial question from the group leaders. Only to a low degree, the group leaders needed to remind the participants that the aim of the study was to reveal possible obstacles to the use of the checklist as intended in clinical practice.

Several interviewees called for more managerial support and anchoring. Concrete points were that the use of checklists must be put into a system, and deviations from checklists must be raised with the employees and included as part of patient safety and quality improvement work.

Managers must ensure that everyone receives training in the use of checklists and that clarification regarding responsibility for completing them is clarified. Although it emerged that managers have a great deal of responsibility, it was also clarified that all employees must develop a sense of responsibility for using checklists and that this involves a change in attitude that must be worked on in all clinics and departments. Safety outcomes, not merely safety processes, and positive staff experiences appear to be relevant to address in future studies, which also is in accordance with emerging opinions in patient safety research.^31^

It emerged that the working style and relationship between the employees and between the employees and their leaders in the sections/departments impacted how and whether checklists for safe surgery were used. The use of checklists was related to the possibilities the employees have to propose other measures regarding the working environment and workflow in the operating departments. Some keywords here were collaboration, patient safety, hierarchy, management, ‘location’ of patient care, etc. It may appear as if the employees are trying to use the ‘checklist problem’ as an aid to express a long-awaited need to address other daily challenges that they find challenging and have a major impact on their everyday working life.

Based on the feedback in the groups, it may indicate that there are many ‘heavy’ challenges in the working environment within this type of workplace and that there are possibilities that, according to the employees, this could affect patient safety. It may appear as if each individual employee’s sense of responsibility concerning the use of checklists is crucial for the implementation to be successful and well looked after.

The responsibility for leading the reading of the checklist in its three different phases should be unanimously described in the procedures used in every single unit. According to our findings, an enhanced focus on the responsibility and fulfilment of all elements of phase three appears to be worth prioritising at least in the units from which our study stems. At one of the hospitals, there was confusion about who should guide through phase 2.

The checklist has to be adjusted to the specific areas of use. Further work should be done to define core elements to be covered in every situation and what type of elements to be tailored to the specific situations. However, even more important is probably that the checklist does not remain a single and isolated safety measure. It should be regarded as one of several instruments that build a safety system. If not, using a checklist may be perceived as futile or even counterproductive to the safety level of services provided.

Some core personnel were occasionally missing at the beginning of the procedure, which could also lead to a lack of fulfilment of the checklist procedure. Uncertainty about where to document the fulfilment of the list in the patient files was also presented as a topic of concern. These factors point to a lack of managerial involvement as a core factor behind the failure to use the checklist as intended. At least, the leaders must follow-up reports on deviations from standards described in the safety and quality system.

The main limitation of this study is that it is restricted to two hospitals in Norway. Some of the participants had experiences from other hospitals too. They could tell that the obstacles in our two settings are well known from other settings too. However, it was claimed by one of the participants that the use of the checklist, in particular outside the operating theatre (e.g. at surgery in outpatient clinics) could vary considerably between hospitals. Still, this participant commented that the obstacles experienced in principle were similar.

Another limitation is the relatively low number of participants, but as saturation was reached quite early, this factor is judged to be of minor importance. In this study, we were not able to reveal differences related to age, sex, or professional background of the participants. Those factors are now followed up through another quantitative study at the same two hospitals.

Lastly, the HazID method used here is developed and tested as an instrument for safety improvement, not primarily as a research method.^27^ However, its similarity with focus group interviews as a research method is fundamental. We therefore find it sensible to use HazID also as a method in case-based research projects.

## Conclusion

Even though the groups comprised participants from different units and specialities, different age levels and a mixture of both women and men, much information emerged based on personal experiences. This may indicate that they had an experience that it was ‘safe’ to open up to the other participants.

One concern of importance for many of the interviewees was the lack of a sense of teamwork and common responsibility for patient safety efforts and, thus, the use of the list.

In general, it appears that the majority of employees believe that checklists are necessary, and they should also be implemented for interventions located outside the operating theatres and for emergency treatments. However, many surrounding factors are perceived as obstacles to its use. The managerial level at every single unit should follow-up on these factors. More knowledge is needed on how implementation and sustained use may be supported by clinical leaders. It obviously is not enough of a managerial decision to establish new and sustainable safety practices.

## References

[1] WHO. The second global patient safety challenge: safe surgery saves lives. Geneva: World Health Organization, 2008.

[2] Haynes AB Weiser TG Berry WR , et al. A surgical safety checklist to reduce morbidity and mortality in a global population. N Engl J Med 2009; 360: 491–499. 19144931 10.1056/NEJMsa0810119

[3] Borchard A Schwappach DLB Barbir A , et al. A systematic review of the effectiveness, compliance, and critical factors for implementation of safety checklists in surgery. Ann Surg 2012; 256(6): 925–933. 22968074 10.1097/SLA.0b013e3182682f27

[4] Bergs J Helings J Cleemput I , et al. Systematic review and meta-analysis of the effect of the World Health Organization surgical safety checklist on postoperative complications. Br J Surg 2014; 101: 150–158. 24469615 10.1002/bjs.9381

[5] Haugen AS Murugesh S Haaverstad R , et al. A survey of surgical team members’ perceptions of near misses and attitudes towards Time Out protocols. BMC Surg 2013; 13: 46. 24106792 10.1186/1471-2482-13-46 PMC3851944

[6] Haugen AS Softeland E Almeland SK , et al. Effect of the World Health Organization checklist on patient outcomes: a stepped wedge cluster randomized controlled trial. Ann Surg 2015; 261(5): 821–828. 24824415 10.1097/SLA.0000000000000716

[7] Ragusa PS Bitterman A Auerbach B , et al. Effectiveness of surgical safety checklists in improving patient safety. Orthopedics 2016: 39(2): e307–e310. 10.3928/01477447-20160301-02 26942472

[8] Papadakis M Meiwandi A Grzybowski A. The WHO safer surgery checklist time out procedure revisited: strategies to optimise compliance and safety. Int J Surg 2019; 69: 19–22. 31310820 10.1016/j.ijsu.2019.07.006

[9] De Espindola S Do Nascimento KC Knihs NS , et al. Safe surgery checklist: content validation proposal for liver transplantation. Revista Brasileira de Enfermagem 2020; 73(suppl 6): e20190538 10.1590/0034-7167-2019-0538 33338123

[10] GlobalSurg Collaborative. Pooled analysis of WHO Surgical Safety Checklist use and mortality after emergency laparatomy. Br J Surg 2019; 106(2): e103–e112. 10.1002/bjs.11051 PMC6492154 30620059

[11] Delisle M Pradarelli JC Panda N , et al. Variation in global uptake of the Surgical Safety Checklist. Br J of Surg 2020; 107(2): e151–e160. 10.1002/bjs.11321 31903586

[12] Eiding H Kongsgaard UE Braarud A-C. Interhospital transport of critically ill patients: experiences and challenges, a qualitative study. Scand J Trauma Resusc Emerg Med 2019; 27(1): 27. 30832699 10.1186/s13049-019-0604-8 PMC6399939

[13] Gundersen KM Heide B Hjukse JB , et al. Implementering av sjekkliste for trakeotomi ved intensivavdelingen på Rikshospitalet. KLoK-oppgave modul 8. Oslo: Universitetet i Oslo, 2019.

[14] Wæhle HV Haugen AS Wiig S , et al. How does the WHO Surgical Safety Checklist fit with existing perioperative risk management strategies? An ethnographic study across surgical specialties. BMC Health Serv Res 2020; 20: 111. 32050960 10.1186/s12913-020-4965-5 PMC7017532

[15] Turley N Elam M Brindle ME. International perspectives on modifications to the surgical safety checklist. JAMA Netw Open 2023; 6(6): e2317183. 10.1001/jamanetworkopen.2023.17183 PMC10248735 37285154

[16] Haugen AS. Modifications of the World Health Organization’s Surgical Safety Checklist—Ways forward to ensure sustainable implementation. JAMA Netw Open 2023; 6(6): e2317100. 10.1001/jamanetworkopen.2023.17100 37285162

[17] Leveson NG. Engineering a safer world. Systems thinking applied to safety. Cambridge, MA: The MIT Press, 2011.

[18] Reason J. Managing the risks of organizational accidents. Farnham: Ashgate, 1997.

[19] PSA Norway. Principles for barrier management in the petroleum industry. Barrier memorandum 2017. Stavanger: Petroleum Safety Authority Norway, 2017.

[20] Clay-Williams R Colligan L. Back to basics: checklists in aviation and healthcare. BMJ Qual Saf 2015; 24: 428–431. 10.1136/bmjqs-2015-003957 PMC4484042 25969512

[21] Perrow C. Normal accidents: living with high-risk technologies. New York, NY: Basic Books, 1984.

[22] Løland MV Braut GS Lichtenberg SM , et al. Tools for establishing a sustainable safety culture within maternity services. A retrospective case study. SAGE Open Med 2023; 11: 20503121231164264. 10.1177/20503121231164264 PMC10071155 37026106

[23] Gerring J. What is a case study, and what is it good for? Am Polit Sci Rev 2004; 98(2): 341–354. www.jstor.org/stable/4145316

[24] Siddiqui NA Nandan A Sharma M , et al. Risk management techniques HAZOP & HAZID study. Int J Occup Health Saf Fire Environ Allied Sci 2014; 1(1): 5–8.

[25] Statens vegvesen. Risikovurderinger i vegtrafikken. Håndbok V721. Oslo: Vegdirektoratet, 2021. https://www.vegvesen.no/globalassets/fag/handboker/hb-v721-risikovurdering.pdf

[26] Aven T Thekdi S. Risk science. An introduction. Abingdon: Routledge, 2022.

[27] Palm M Moen L Braut GS. Risk analyses in the intersection between patient and workplace safety: a case study of hazids in para-clinical supporting systems in specialized health care. SAGE Open Med 2020; 8: 2050312120977136. 10.1177/2050312120977136 PMC7705811 33294187

[28] NESH. Guidelines for research ethics in the social sciences and the humanities. 5th ed. Oslo: National Committee for Research Ethics in the Social Sciences and the Humanities, 2021.

[29] GDPR. (2016). General Data Protection Regulation. Regulation (EU) 2016/679. Official Journal of the European Union.

[30] Strauss A Corbin J. (1998). Basics of qualitative research: techniques and procedures for developing grounded theory. 2nd ed. Thousand Oaks, CA: SAGE, 1998.

[31] Finn M Walsh A Rafter N , et al. Effect of interventions to improve safety culture on healthcare workers in hospital settings: a systematic review of the international literature. BMJ Open Quality 2024; 13: e002506. 10.1136/bmjoq-2023-002506 PMC11086522 38719514

